# Analysis of Spatiotemporal Evolution Characteristics and Driving Factors of NPP in Guizhou Province, China

**DOI:** 10.1002/ece3.72231

**Published:** 2025-10-02

**Authors:** Bo Xie, Yi Liu, Han Fan, Mingming Zhang

**Affiliations:** ^1^ College of Forestry Guizhou University Guiyang China; ^2^ Guizhou Provincial Wetland and Public Welfare Forest Conservation Center Guiyang China; ^3^ Research Center for Biodiversity and Nature Conservation of Guizhou University Guiyang China

**Keywords:** geographic detector, karst region, net primary productivity, spatiotemporal variation

## Abstract

Karst ecosystems are ecologically fragile and highly sensitive to climate change. This study explored the spatiotemporal changes and driving mechanisms of net primary productivity (NPP) in Guizhou Province, a typical karst region in Southwest China, from 2000 to 2020. By integrating multisource geospatial datasets, we employed comprehensive spatiotemporal analytical methods including Sen + Mann‐Kendall trend analysis, Hurst index, and coefficient of variation, complemented by correlation analysis and the geographic detector modeling. Key findings reveal: (1) A significant upward trend in NPP with an interannual variation of 3.65 g C m^−2^ a^−1^ and a multiyear average of 785.47 g C m^−2^ a^−1^; (2) Distinct spatial heterogeneity showing southern high‐value zones contrasting with lower values in northern, eastern, and western regions, with 45.76% of areas demonstrating significant positive NPP trends; the overall volatility is stable; (3) Driving mechanism analysis identifies precipitation, soil moisture, and population density as dominant factors, with anthropogenic influences exhibiting increasing temporal dominance.

## Abstract 2

This study analyzed and quantified the spatiotemporal dynamics and driving mechanisms of vegetation net primary productivity in ecologically fragile karst areas, hoping to provide a scientific reference for regional ecological protection and sustainable socioeconomic development.

## Graphical abstract

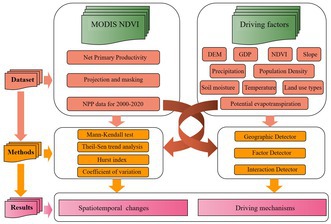

## 1 Introduction

Climate change is triggering ecological responses and adaptive pressures at a global scale (Mi and Zhang 2023). Karst ecosystems, recognized as ecologically vulnerable regions, demonstrate heightened susceptibility to environmental perturbations (Zhang et al. 2023; He et al. 2022). These ecosystems are characterized by pronounced landscape fragmentation, diminished landscape structural stability, and enhanced spatial heterogeneity (Feng et al. 2014; Liu, Zhang, et al. 2022), resulting in a high degree of spatiotemporal heterogeneity in vegetation change (Huang et al. 2022). Analyzing the spatiotemporal dynamics of NPP and quantifying their driving factors hold critical implications for formulating conservation strategies, ecological restoration practices, and sustainable development in fragile ecosystems (Liu, Li, et al. 2018; Xu et al. 2024).

As a core part of terrestrial ecosystems, vegetation performs indispensable ecological functions through regulating material and energy fluxes, maintaining climatic homeostasis, and supporting sustainable development (Liu, Zhang, et al. 2018; Xie et al. 2023). NPP is a pivotal biophysical indicator characterizing vegetation's photosynthetic carbon sequestration efficiency, which refers to the remaining portion after green plants, through photosynthesis per unit time and unit area, capture solar energy and synthesize the total amount of organic matter, minus the energy consumed by their own respiration (Field et al. 1998; Roxburgh et al. 2005). It is a key indicator of ecosystem health and productivity, and a direct tool for assessing carbon sequestration capacity and ecological environmental change (Teng et al. 2020; Yue et al. 2022). While conventional ground‐based observation methods enable high‐precision NPP estimation, their spatiotemporal scalability remains constrained by sampling costs and scale‐dependent limitations; in contrast, the integration of multisource remote sensing data with geospatial technologies provides an efficient solution for continuous monitoring across regional to global domains (Field et al. 1995; Goetz et al. 1999; Zhu et al. 2005; Li et al. 2020). Currently, the Google Earth Engine (GEE) has a large number of global geoscience datasets (Zhang, Hu, et al. 2021), which can efficiently process remote sensing big data and provide effective support for vegetation remote sensing monitoring (Zhou et al. 2025). The annual NPP product obtained based on the GEE platform has a resolution of 500 m × 500 m, and the data simulation accuracy is high, and its accuracy has been verified (Wang, Tang, et al. 2022). It has been widely used in NPP research (Lü et al. 2017; Gulbeyaz et al. 2018; Chen et al. 2023).

In recent years, the research on the spatiotemporal differentiation mechanism and driving factors of NPP in karst areas has achieved rich results. Previous studies revealed a fluctuating upward trend in vegetation coverage across southwest China's karst regions (Cai et al. 2014; Zhang et al. 2023). The spatiotemporal pattern of NPP is synergistic and complex due to multiscale drivers (Wu et al. 2014), with climate sensitivity being universally identified as a critical determinant of NPP dynamics, where vegetation changes have been predominantly attributed to climatic variations (Ma et al. 2022; He et al. 2022; Xu et al. 2022). However, the role of climatic factors is not linear; excessive temperature inhibits vegetation development by enhancing evapotranspiration (Zhou et al. 2022), and precipitation beyond optimal thresholds diminishes NPP growth potential (Qian et al. 2023). In addition, natural processes such as the fertilization effect of CO_2_ (Maschler et al. 2022), solar radiation (Ma et al. 2024), and extreme weather (Wang et al. 2015) also profoundly affect the dynamics of NPP. Human activities demonstrate dualistic impacts. Negative impacts include intensive anthropogenic disturbances exacerbating soil degradation in karst fault basins, thereby inhibiting vegetation growth and reducing coverage (Xu et al. 2019). The positive impact means that the implementation of ecological restoration projects can effectively promote the increase of NPP (Hong et al. 2021). Therefore, the spatiotemporal dynamics of NPP are affected by the multiple coupling effects of climatic factors, topographic and geological conditions, and human activities. Traditional analytical frameworks based on linear constraint assumptions and factor independence hypotheses prove inadequate in disentangling these complex surface processes, potentially introducing analytical biases (Li et al. 2022; Huang et al. 2023). Correlation, regression analysis, and residual trend analysis are all based on the assumption that there is a significant linear relationship between the driving forces and vegetation change (Yin et al. 2017). While regression methods elucidate linear relationships, the relationship between vegetation and driving factors is not simply linear due to the complex response of vegetation growth to driving variables (Pan et al. 2018). Consequently, the results obtained may not fully capture the actual driving relationships (Xue et al. 2023). The geographical detector methodology, designed for spatial heterogeneity quantification and driving mechanism exploration, offers a paradigm shift by overcoming linear limitations inherent in conventional statistical approaches (Wang and Xu 2017).

Guizhou Province is a unique geographical unit; the karst landform is widely developed, and the ecological environment is extremely fragile (Huang et al. 2018). Systematic ecological restoration measures have been carried out in the region since the 21st century, such as the project of returning farmland to forests and grasslands, the conservation plan of natural forest resources, and the comprehensive control of rocky desertification (Tong et al. 2016; Xiong et al. 2012; Shao et al. 2022). Concurrently, the Western Development Strategy initiated in 2000 (Zhuo and Deng 2020) has driven rapid economic growth and urbanization in Guizhou, profoundly altering land surface configurations and intensifying pressures on ecosystem integrity (Wang, Zhang, et al. 2022). Based on this, our objectives were to (1) quantitatively characterize the spatiotemporal variation of NPP across Guizhou over the past 2 decades, and (2) disentangle the driving mechanisms of natural and anthropogenic drivers on NPP dynamics. The findings are expected to inform dual‐objective strategies for ecological conservation and sustainable socioeconomic development in fragile karst ecosystems.

## 2 Materials and Methods

### 2.1 Study Area

**FIGURE 1:**
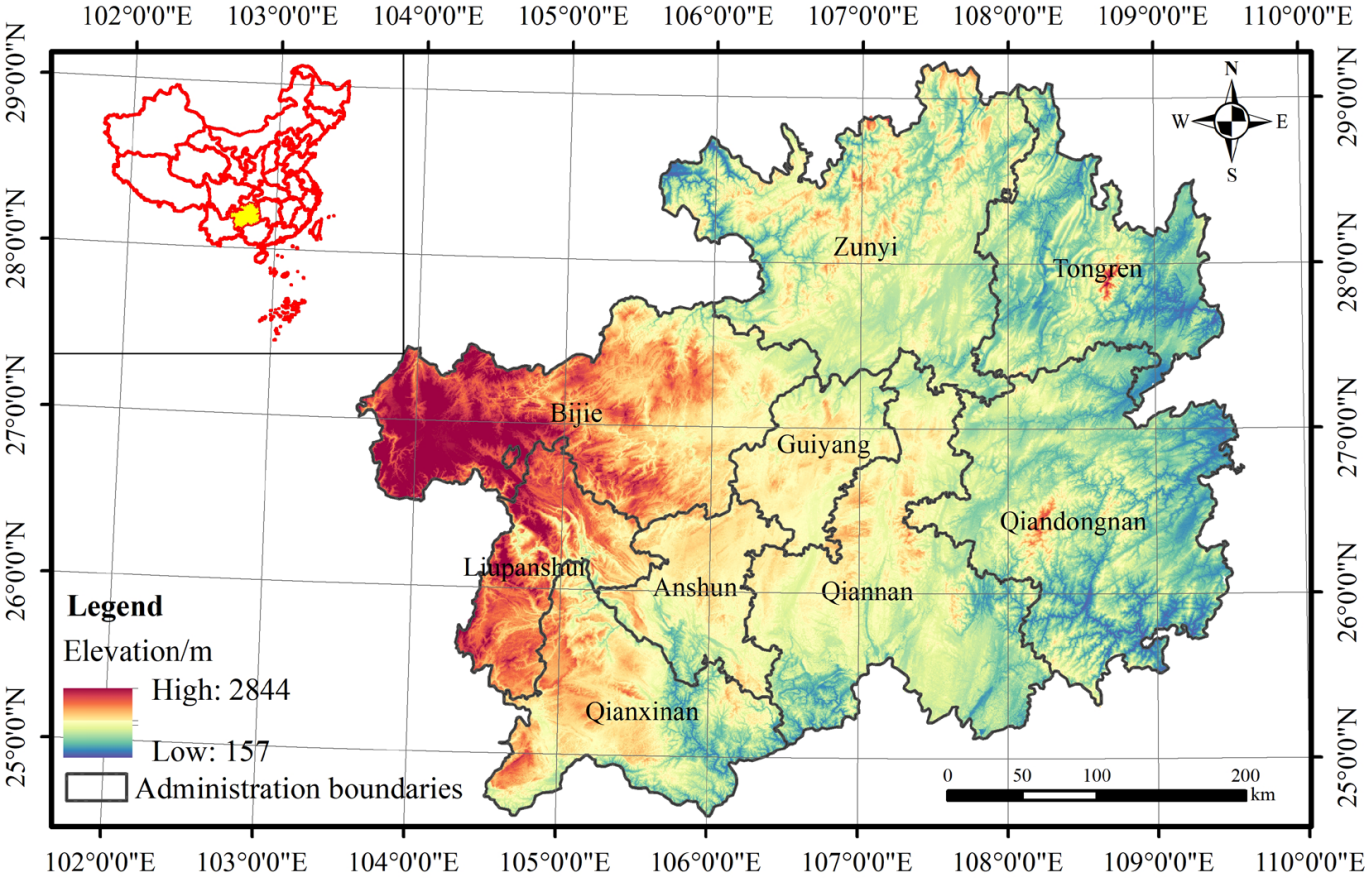
Location of study region.

Guizhou Province is situated in the Yunnan‐Guizhou Plateau in Southwest China, its geographical range of 24°37′–29°13′ N, 103°36′–109°35′ E (Figure 1). It comprises six municipal districts and three ethnic autonomous prefectures, covering a total land area of 176.20 km^2^. The topography descends from west to east with elevational variations between 157 m and 2844 m. Guizhou Province has a typical subtropical monsoon climate, with the mean annual temperature oscillating between 10°C and 18°C and annual precipitation averaging 1000–1500 mm. Guizhou Province is a typical karst distribution area; over 70% of its territory is characterized by carbonate rock formations, causing a vulnerable ecosystem.

### 2.2 Data Resources

NPP data were sourced from MOD17A3HGF (http://glovis.usgs.gov/), with a spatial resolution of 500 m and a temporal resolution of 16 days. The time series covers the period 2000–2020.

Temperature, precipitation, and soil moisture data, with a spatial resolution of 1 km, and NDVI data, with a spatial resolution of 250 m, were all obtained from the Tibetan Plateau Data Center (https://data.tpdc.ac.cn). Potential evapotranspiration data was sourced from the National Earth System Science Data Center (https://www.geodata.cn), with a spatial resolution of 1 km. The DEM (Digital Elevation Model) utilized GDEMV3 30 m resolution digital elevation data (https://www.gscloud.cn/), from which slope was derived. Land‐use data has a resolution of 30 m, while GDP and population density data have a resolution of 1 km; all were acquired from the Resource and Environment Science and Data Center (https://www.resdc.cn/). All datasets underwent a unified projection coordinate system conversion and were resampled to a spatial resolution of 500 m.

Table 1 shows the datasets applied in this study, including data name, unit, resolution, and data link.

**TABLE 1:** Details of the dataset resources applied in this study.

Data	Unit	Spatial resolution	Data resources
NPP	g·C/m^2^	500 m	MOD17A3HGF (https://lpdaac.usgs.gov/)
DEM	m	30 m	Geospatial data cloud (http://www.gscloud.cn)
Temperature	°C	1 km	The National Tibetan Plateau Data Center (https://data.tpdc.ac.cn/)
Precipitation	mm	1 km	
Soil moisture	mm/m^3^	1 km	
NDVI	—	250 m	
Potential evapotranspiration	mm	1 km	National Earth System Science Data Center (https://www.geodata.cn)
Gross domestic product (GDP)	yuan/km^2^	1 km	Resource and Environment Science and Data Center (http://www.resdc.cn/)
Land‐use types	—	30 m	
Population Density	person/km^2^	1 km	

### 2.3 Methods

#### 2.3.1 Theil–Sen + Mann–Kendall Trend Analysis

The Theil–Sen + Mann–Kendall trend analysis method does not need the data to obey a specific distribution, is not affected by missing values and outliers, and also has a solid theoretical basis in the significance level test, and the results are relatively objective (Jiang et al. 2020; Shen et al. 2022; Wei, Yang, et al. 2022). The formula is:

(1) $$\text{Slope}=\frac{n\times \sum_{n=1}^{n}\left(i\times {\mathrm{NPP}}_{i}\right)-\sum_{n=1}^{n}i\sum_{n=1}^{n}{\mathrm{NPP}}_{i}}{n\times \sum_{i=1}^{i}i^{2}-{\left(\sum_{i=1}^{n}i\right)}^{2}}$$

where *slope* represents the change trend; *NPP*_*i*_ is the NPP value of year *i*; *n* represents time in the study period, *Slope* > 0 indicates NPP has an upward trend; *Slope* < 0 indicates NPP has a downward trend.

(2) $$z=\left\{\begin{array}{c} \frac{S-1}{\sqrt{\mathrm{var}\left(S\right)}},S>0;\\ \;0\;,S=0;\\ \frac{S+1}{\sqrt{\mathrm{var}\left(S\right)}},S>0; \end{array}\right.$$

(3) $$S=\sum_{i=n}^{n-1}\sum_{j=i+1}^{n}\mathrm{sign}\left({\mathrm{NPP}}_{j}-{\mathrm{NPP}}_{i}\right)$$

(4) $$s\mathrm{gn}=\left({\mathrm{NPP}}_{j}-{\mathrm{NPP}}_{i}\right)=\left\{\begin{array}{c} 1,{\mathrm{NPP}}_{j}-{\mathrm{NPP}}_{i}>0\\ 0,{\mathrm{NPP}}_{j}-{\mathrm{NPP}}_{i}=0\\ -1,{\mathrm{NPP}}_{j}-{\mathrm{NPP}}_{i}<0 \end{array}\right.$$

(5) $$\mathrm{Var}\left(S\right)=\frac{n\left(n-1\right)\left(2n+5\right)}{18}$$

where *NPP*_*i*_ and *NPP*_*j*_ denote the *NPP* values for years *i* and *j*, respectively, *n* represents time in the study period. A two‐sided trend test is used when |*Z*| ≤ *Z*1−α/2, indicating that the sequence does not trend significantly at the α level, and when |*Z*| > *Z*1−α/2, indicating that the sequence is trending significantly at the α level. Please check for details in Table 2.

**TABLE 2:** Driving factors of the NDVI.

Factor type	Specific indicators
Natural factors	DEM
	Temperature
	Precipitation
	Soil moisture
	NDVI
	Potential evapotranspiration
Human activities	Gross domestic product (GDP)
	Land‐use types
	Population Density

#### 2.3.2 Coefficient of Variation (Cv)

CV is an indicator to describe the dispersion degree of long‐term series data, which can be used to indicate the stability and fluctuation degree of vegetation NPP (Liu, Zhang, et al. 2022). The formula is:

(6) $$\mathrm{CV}=\frac{1}{\bar{x}}\sqrt{\frac{\sum_{i=1}^{n}{\left(x_{i}-\bar{x}\right)}^{2}}{n-1}}$$

where CV denotes the coefficient of variation; *x*_*i*_ is the NPP value for year i; $\bar{x}$ is the average value of NPP from 2000 to 2020; *n* represents time in the study period. Divide the CV value into: high stability (CV < 0.05), relatively high stability (0.05 ≤ CV < 0.1), moderate volatility (0.1 ≤ CV < 0.15), relatively high volatility (0.15 ≤ CV < 0.2), and high volatility (CV ≥ 0.2).

#### 2.3.3 Hurst Index

The Hurst exponential method is built based on the R/S analysis and is applied to quantify the degree of persistent dependence in a time series, which is proposed by Harold Edwin Hurst (Jiang et al. 2017; Liu, Zhang, et al. 2022; Yang, Sun, et al. 2021). Assume a time series {*NPP*_*t*_, *t* = 1, 2, …, *N*}, take a sequence τ = 1, 2, …, *N*, define the mean series for τ:

(7) $$N\left(\tau \right)=\frac{1}{\tau }\sum_{t=1}^{\tau }{\mathrm{NPP}}_{\tau },\tau =1,2,$$

Cumulative deviation sequence:

(8) $$X\left(t,\tau \right)=\sum_{t=1}^{\tau }\left({\mathrm{NPP}}_{t}-{\bar{\mathrm{NPP}}}_{\tau }\right),1\leq t\leq \tau$$

Range sequence:

(9) $$R\left(\tau \right)=\max_{1\leq t\leq \tau }X\left(t,\tau \right)-\min_{1\ll t\ll \tau }X\left(t,\tau \right)$$

Standard deviation sequence:

(10) $$S\left(\tau \right)=\sqrt{\left[\frac{1}{\tau }\sum_{t=1}^{\tau }{\left({\mathrm{NPP}}_{t}-{\bar{\mathrm{NPP}}}_{\tau }\right)}^{2}\right],\tau =1,2,\ldots }$$

Calculate H

(11) $$\frac{R\left(\tau \right)}{S\left(\tau \right)}={\left(c\times \tau \right)}^{H}$$

where H denotes the Hurst exponent, *c* is a constant, and *τ* is the sequence length. If H < 0.5, the sequence is reversed in the future; if H = 0.5, the series is independent of each other; if 0.5 < H < 1, the series is positive in the future, and the past trend will be continued. By overlaying the Hurst index and Theil–Sen trend analysis results in the ArcGIS Raster Calculator, we derived four persistence categories of NPP dynamics: Persistent degradation, Degradation to improvement, Persistent improvement, Improvement to degradation.

#### 2.3.4 Correlation Analysis

Correlation analysis eliminates the influence of other variables, allowing for an accurate exploration of the relative relationship between two variables (Jiang et al. 2020). Using the method to analyze the degrees of climatic factors impacting on NPP. The formula is:

(12) $$R=\frac{\sum_{i=1}^{n}\left(y_{i}-\bar{y}\right)\left(x_{i}-\bar{x}\right)}{\sqrt{\sum_{i=1}^{n}{\left(y_{i}-\bar{y}\right)}^{2}\times \sum_{i=1}^{n}{\left(x_{i}-\bar{x}\right)}^{2}}}$$

where *R* is the correlation coefficient, *n* represents time in the study period, *y*_*i*_ represents the NPP value for year *i*, $\bar{y}$ represents average NPP value, $x_{i}$ represents the value of a certain driver for year *i*, $\bar{x}$ represents average value of a certain driver.

#### 2.3.5 Geographic Detector

The geographical detector, as a pivotal methodological tool for spatial heterogeneity analysis, is fundamentally grounded in the theory of spatial stratified heterogeneity (Wang et al. 2010). Traditional geographical detectors face limitations in subjectivity and suboptimal discretization methods. To address these constraints, select the parameter‐optimized geographical detector, which transcends the linear assumptions of conventional statistical methods and effectively identifies spatial differentiation drivers without presupposing functional relationships between variables. Leveraging the GD package in R, this research quantitatively evaluates the impacts of diverse driving factors on NPP spatial heterogeneity during 2000–2020 (Table 2). The formula is as follows:

(13) $$q=1-\frac{\sum_{h=1}^{L}\left(N_{h}\delta_{h}^{2}\right)}{N\delta^{2}}$$

where *q* is the factor explanatory force, the value is 0–1; the greater the value of *q*, the greater the corresponding influence is on the number of layers. *N*_*h*_ and *N* are the number of units for *h* and the entire region, respectively. *σ* ^*2*^ is the variance of the indicator.

## 3 Results

### 3.1 Interannual Variation in NPP

**FIGURE 2:**
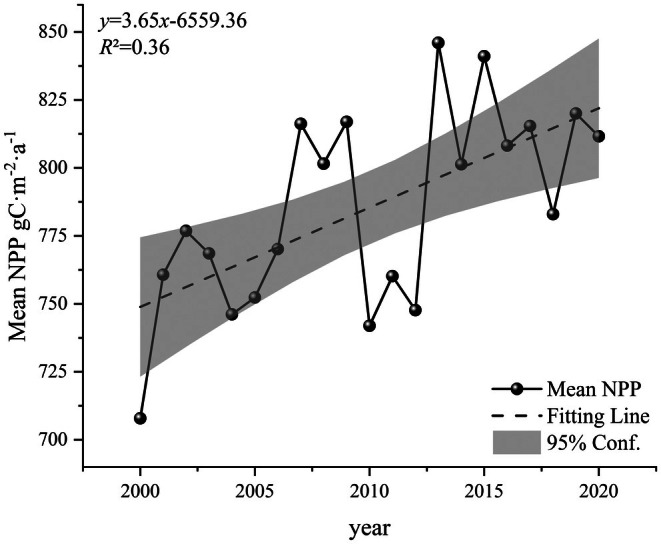
Annual mean NPP variation in Guizhou Province.

During the period 2000–2020, the annual average NPP in Guizhou Province ranged from 707.89 to 845.99 g C m^−2^ a^−1^, with the minimum values recorded in 2000 and the maximum observed in 2015. The interannual variability exhibited a fluctuating upward trajectory (Figure 2), showing a statistically significant growth rate of 3.65 g C m^−2^ a^−1^ (*p* < 0.05), while the multiyear average stabilized at 785.47 g C m^−2^ a^−1^. Notably, the most pronounced NPP increase occurred between 2012 and 2013, with an increment of 98.31 g C m^−2^ a^−1^, whereas the sharpest decline was observed during 2009–2010, registering a reduction in 74.99 g C m^−2^ a^−1^.

### 3.2 Spatial Pattern Variability of NPP

#### 3.2.1 Spatiotemporal Pattern and Characteristics of NPP

**FIGURE 3:**
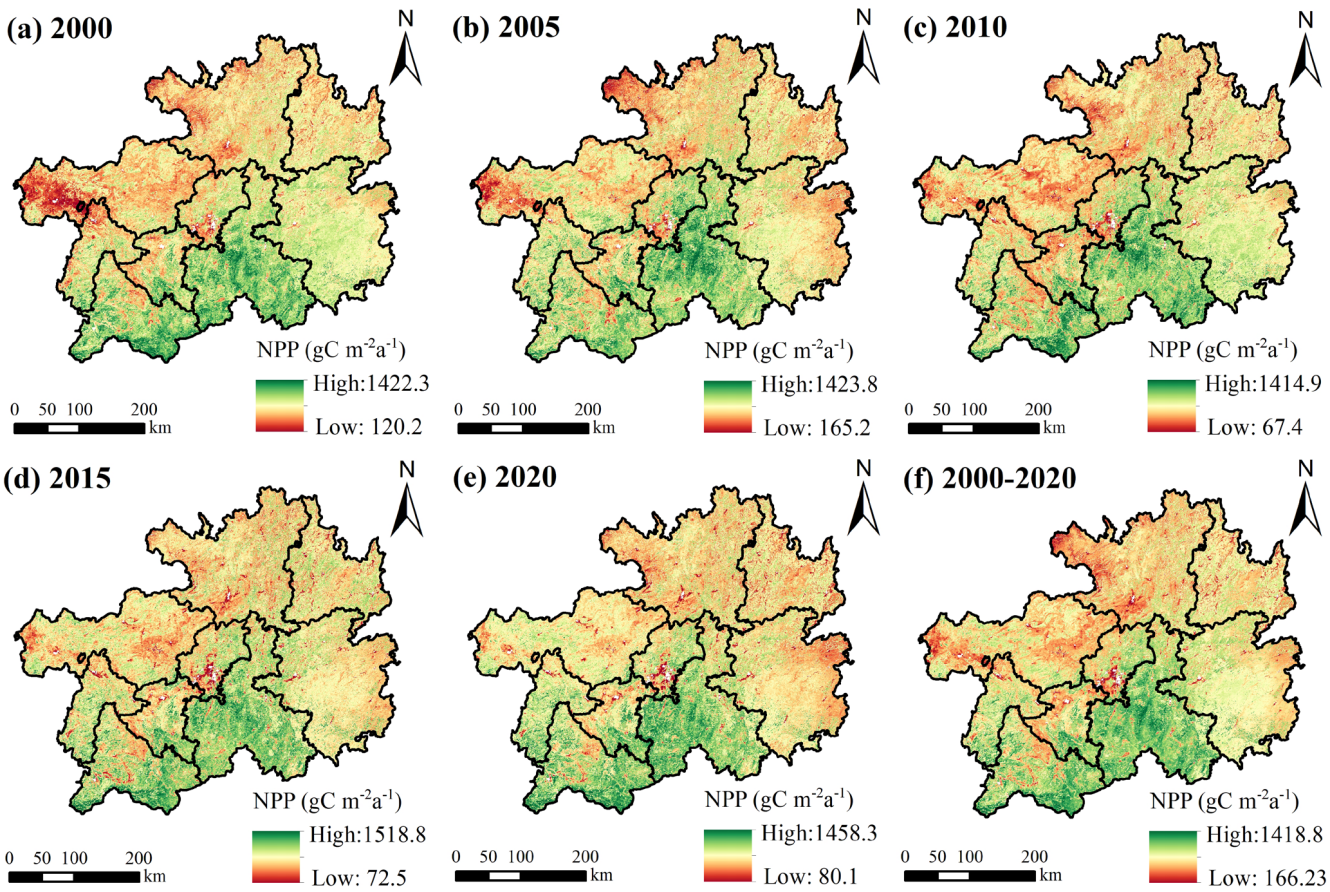
Spatial pattern of NPP in Guizhou Province from 2000 to 2020.

The spatial patterns of annual mean NPP in Guizhou Province are illustrated in Figure 3. The multiyear average NPP exhibited marked spatial heterogeneity, with higher levels predominantly distributed across southern regions, contrasting sharply with lower values in northern, eastern, and western territories. Administratively, high‐value NPP zones were predominantly located in the southern parts of Qiannan Prefecture and Qianxinan Prefecture, while low NPP clusters concentrated in Guiyang City, the southern and northwestern sectors of Zunyi City, and western Bijie City. Notably, Tongren City and Qiandongnan Prefecture displayed no statistically significant high‐ or low‐value NPP zones.

#### 3.2.2 Trend Analysis for NPP

**FIGURE 4:**
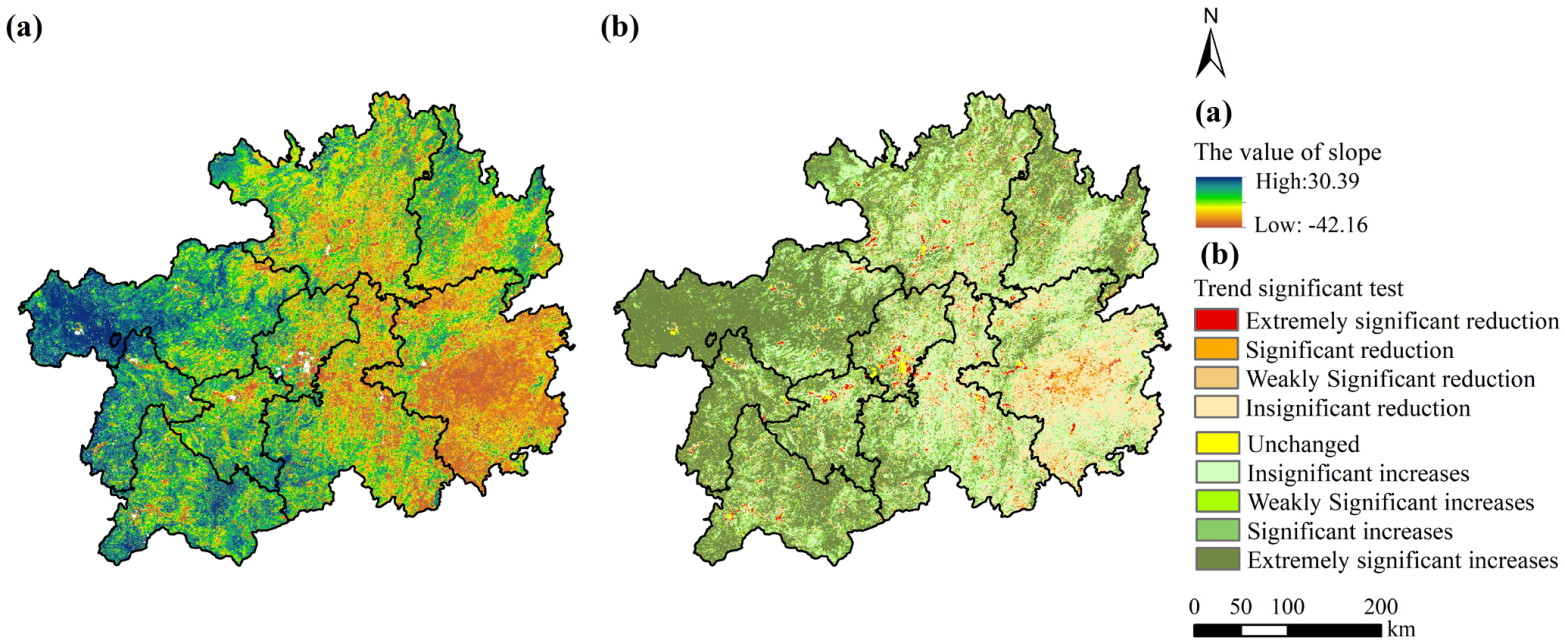
Spatial variation trend and significance test of NPP from 2000 to 2020.

The fitted slope of NPP ranged from 42.16 to 30.39 g C m^−2^ a^−1^, with 78.48% of regions exhibiting positive slopes, indicative of a predominant upward trend (Figure 4a). Spatially, NPP increases mainly in the western, southwestern, and northeastern sectors, while declines predominated in the central and southeastern regions (Figure 4a). Trend significance analysis (Figure 4b, Table 3) revealed that 6.4% of regions showed statistically significant reduction (encompassing Extremely significant reduction, Significant reduction, and Weakly Significant reduction), primarily clustered in the southeastern, central, and northern areas. Conversely, 45.76% of the area displayed significant increases (including Extremely significant increases, Significant increases, and Weakly Significant increases), predominantly distributed in the western, southwestern, and northeastern zones. Collectively, these findings demonstrate that NPP dynamics in Guizhou Province during 2000–2020 were dominated by positive trajectories, reflecting strengthened ecosystem stability over the 20‐year observation period.

**TABLE 3:** Significance test statistics for the trends in NPP.

Trend	Significance of NPP trend	*Z* value	Area/km^2^	Percentage/%
< 0	Extremely significant reduction	2.58 < *Z*	3766	2.14
	Significant reduction	1.96 < *Z* ≤ 2.58	4792	2.72
	Weakly Significant reduction	1.65 < *Z* ≤ 1.96	2713	1.54
	Insignificant reduction	*Z* ≤ 1.65	25827.75	14.66
=0	Unchanged	*Z*	3658.25	2.08
> 0	Extremely significant increases	*Z* > 2.58	45394.5	25.77
	Significant increases	2.58 ≥ *Z* > 1.96	10,540	5.98
	Weakly Significant increases	1.96 ≥ *Z* > 1.65	24,678	14.01
	Insignificant increases	1.65 ≥ *Z*	54750.75	31.09

#### 3.2.3 Cv of NPP

**FIGURE 5:**
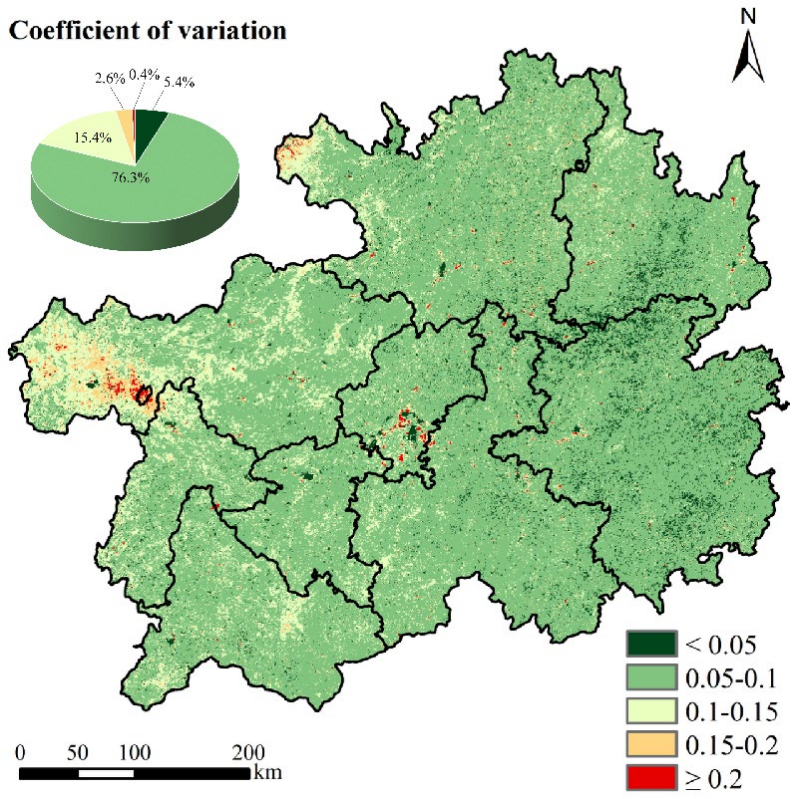
Stability analysis of NPP and the area ratio of each classification.

The CV index for NPP spanned a spatial range of 0–0.81 g C m^−2^ a^−1^, with a mean value of 0.04 g C m^−2^ a^−1^, indicating relatively stable interannual variability. High volatility (CV ≥ 0.2) collectively accounted for 18.3% of the study region, predominantly clustered in the western, central, and northwestern areas of Guizhou Province. Conversely, high stability (CV < 0.05) and relatively high stability (0.05 ≤ CV < 0.1) constituted 81.7% of the area, with high stability zones sporadically distributed in the eastern region (Figure 5). These results demonstrate that NPP fluctuations during the observation period were characterized by predominantly relatively high stability.

#### 3.2.4 Hurst of NPP

**FIGURE 6:**
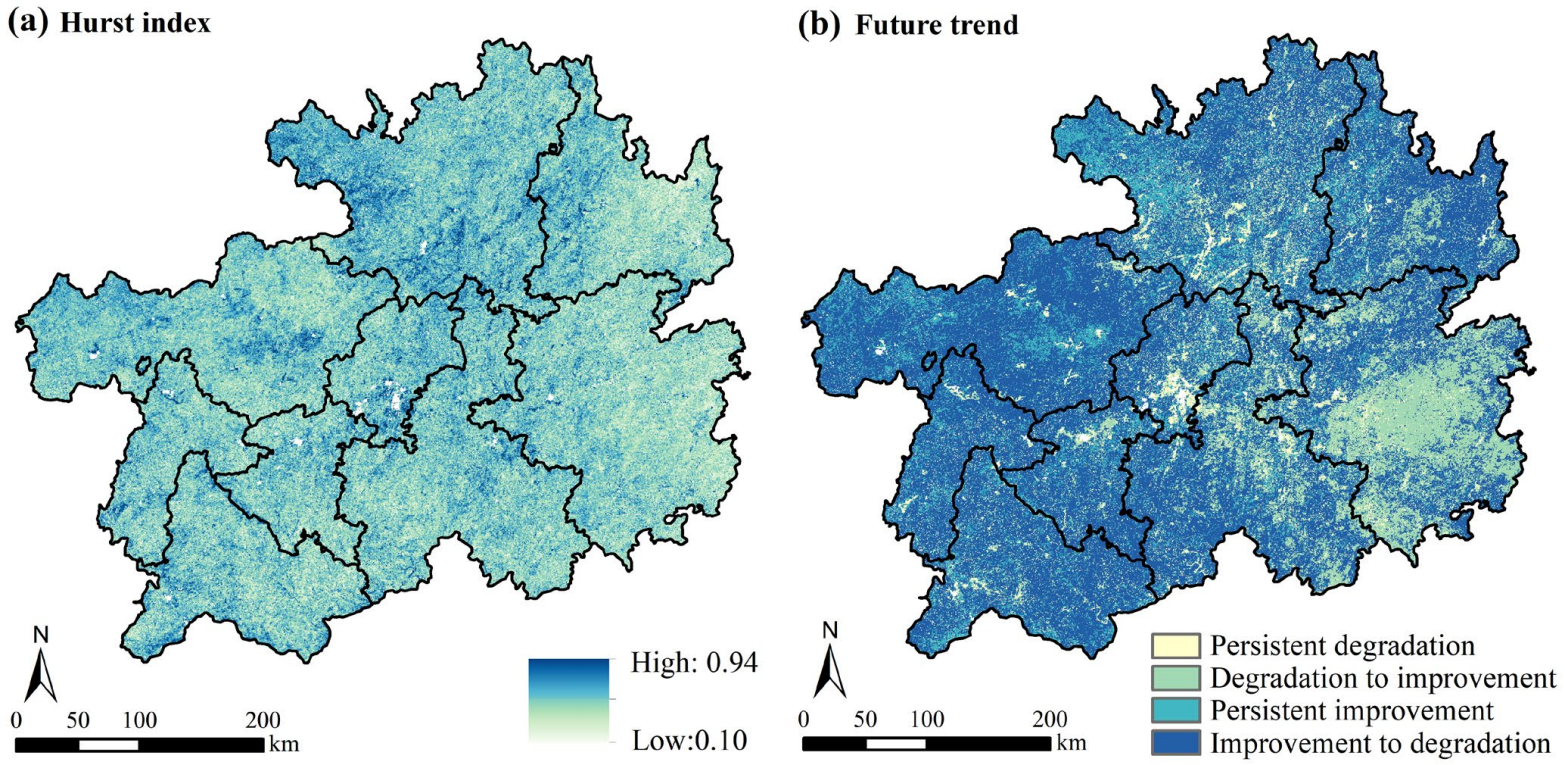
Hurst spatial distribution of the Hurst index (a) and future trends (b).

During 2000–2020, the Hurst index is between 0.10 and 0.94 (Figure 6a), with a mean of 0.44; it indicates that the overall trend in the region is not very persistent. Regions with H < 0.5 accounted for 76.76% of the total area. The results indicate that the average annual NPP in Guizhou Province may show an opposite trend in the future, while H > 0.5 zones constituted 23.24%, indicating that the annual NPP in the region has a significant sustainability trend.

Based on the results of Hurst index and Sen trend overlay analysis, it was revealed that 61.56% of the area is likely to shift from improvement to degradation, 6.3% to persist in degradation, 16.93% to maintain improvement, and 15.20% to transition from degradation to improvement (Figure 6b). Spatially, the future changes of NPP are mainly from improvement to degradation, with persistent degradation mainly distributed in the central and northern regions, and the areas of degradation to improvement are mainly in the southeast, while the areas of persistent improvement are distributed in the northwest.

### 3.3 Drivers of the NPP Change

#### 3.3.1 Meteorological Factors

**FIGURE 7:**
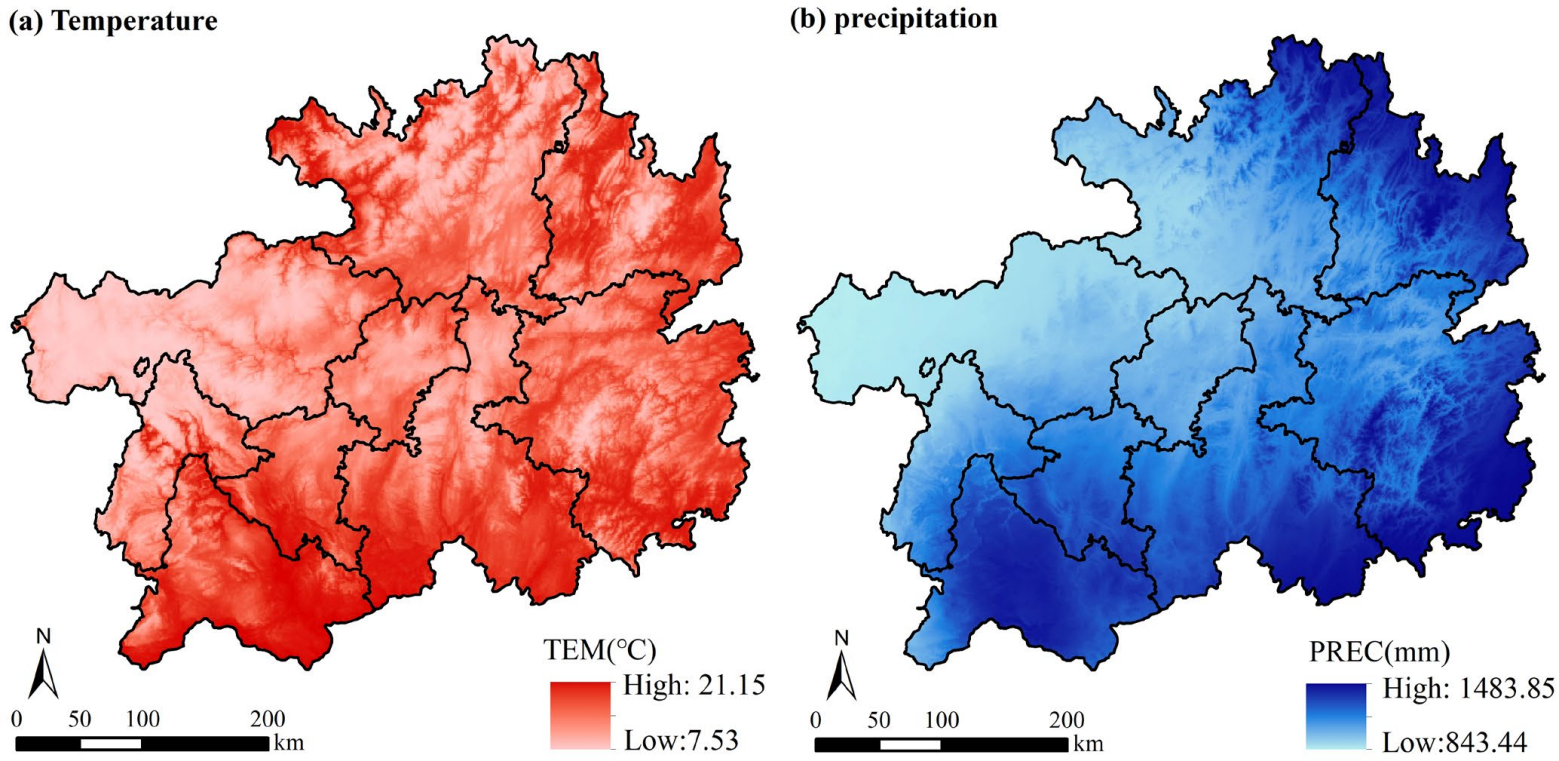
Spatial distribution of mean annual temperature (a) and precipitation (b).

**FIGURE 8:**
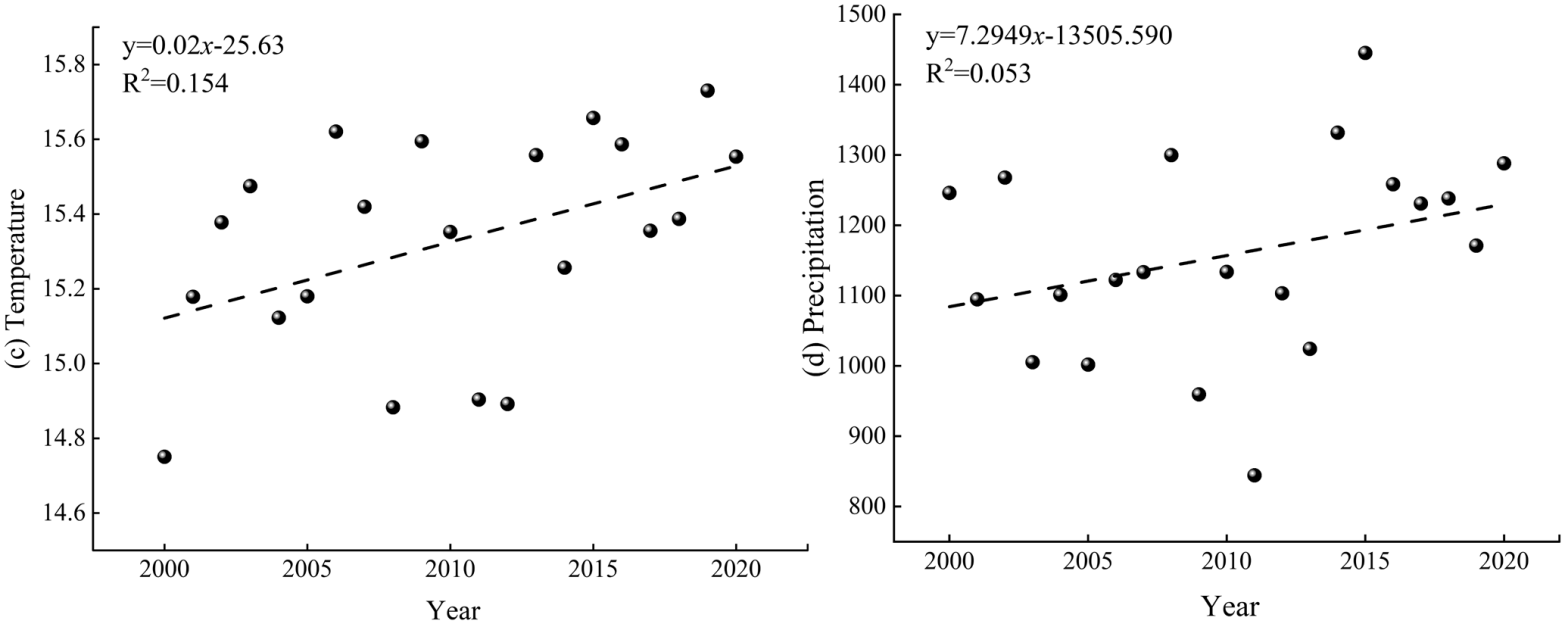
Mean annual temperature (c) and precipitation (d) time trends.

During the study period, the annual average temperature presented a fluctuating increase, with a growth rate of 0.02°C/a, and the spatial distribution of temperature exhibited a south‐high and west‐low pattern (Figures 7a and 8c). The annual average precipitation demonstrated a fluctuating increasing trend, with an annual precipitation of 1157.20 mm and a growth rate of 7.3 mm/a. Specifically, precipitation decreased significantly and was below average during the periods 2003–2007 and 2009–2013, and the spatial pattern exhibited significantly higher levels in the southern and eastern regions of Guizhou Province than in the central and western areas (Figures 7b and 8d).

**FIGURE 9:**
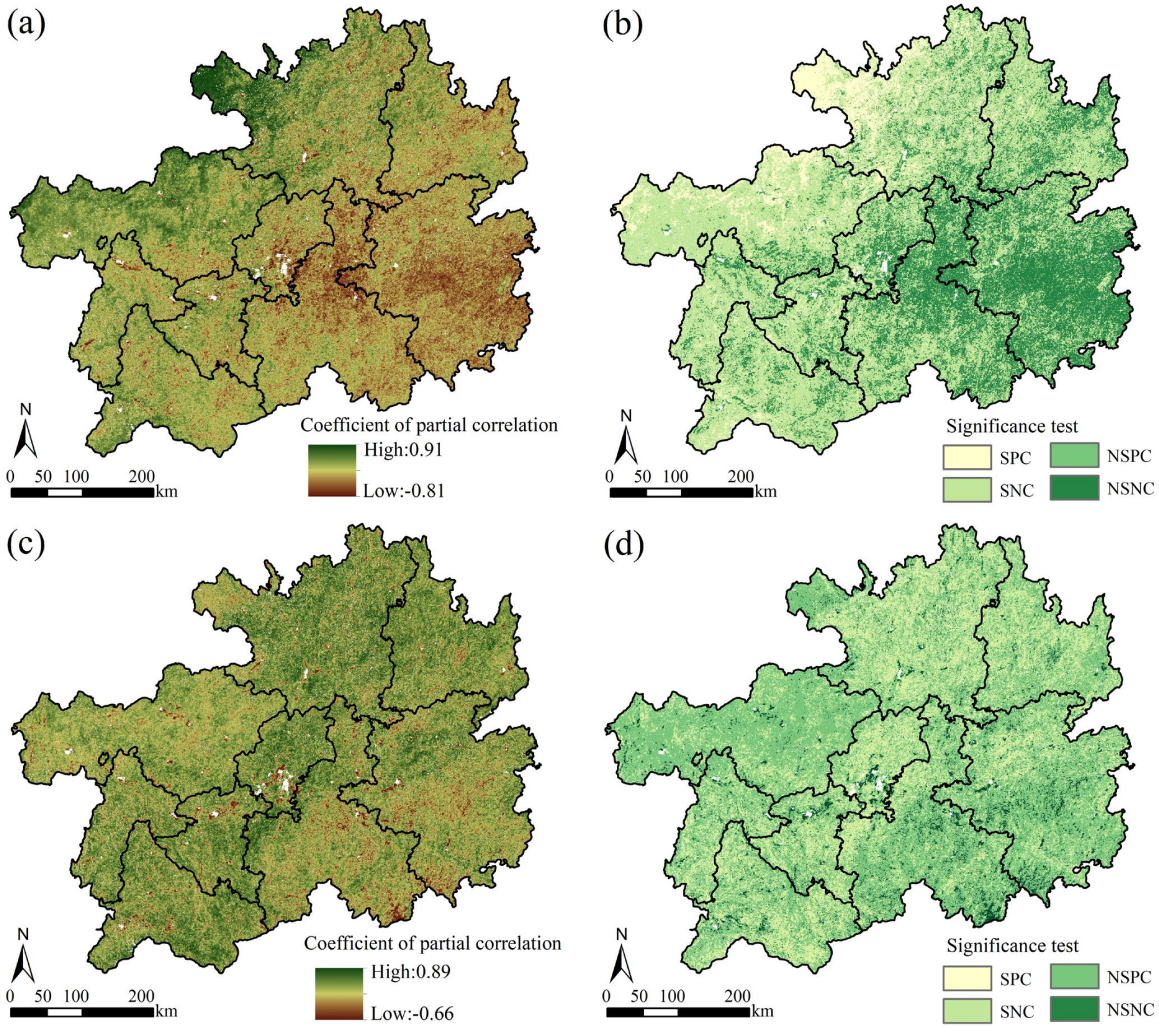
Correlation and significance tests between NPP and precipitation and temperature.

Correlation analysis revealed marked spatial divergence in the relationship between NPP and climatic variables. The correlation coefficients between temperature and NPP spanned 0.66 to 0.89 (Figure 9a), with 94.12% of the study area showing significant temperature influences. Notably, 34.43% of regions showed significant positive correlations, primarily clustered in the eastern, southern, and northern sectors, while 5.88% displayed significant negative correlations, distributed in central and southern zones (Figure 9b, Table 4). For precipitation–NPP interactions, partial correlation coefficients spanned 0.81 to 0.91 (Figure 9c). Areas with significant precipitation impacts accounted for 68.35%, of which 10.7% demonstrated robust positive correlations, primarily distributed in the northern and western areas (Figure 9d, Table 4).

**TABLE 4:** Proportion of correlation analysis area between NPP and meteorological factors/%.

Correlation	Correlation of temperature	Correlation of precipitation
Significant positive correlation SPC (*p* < 0.01)	34.43	10.07
No significant positive correlation NSPC (*p* > 0.05)	59.69	58.28
Significant negative correlation SNC (*p* < 0.01)	0.04	0.96
No significant negative correlation NSNC (*p* > 0.05)	5.84	30.69

#### 3.3.2 Topographic Factors

**FIGURE 10:**
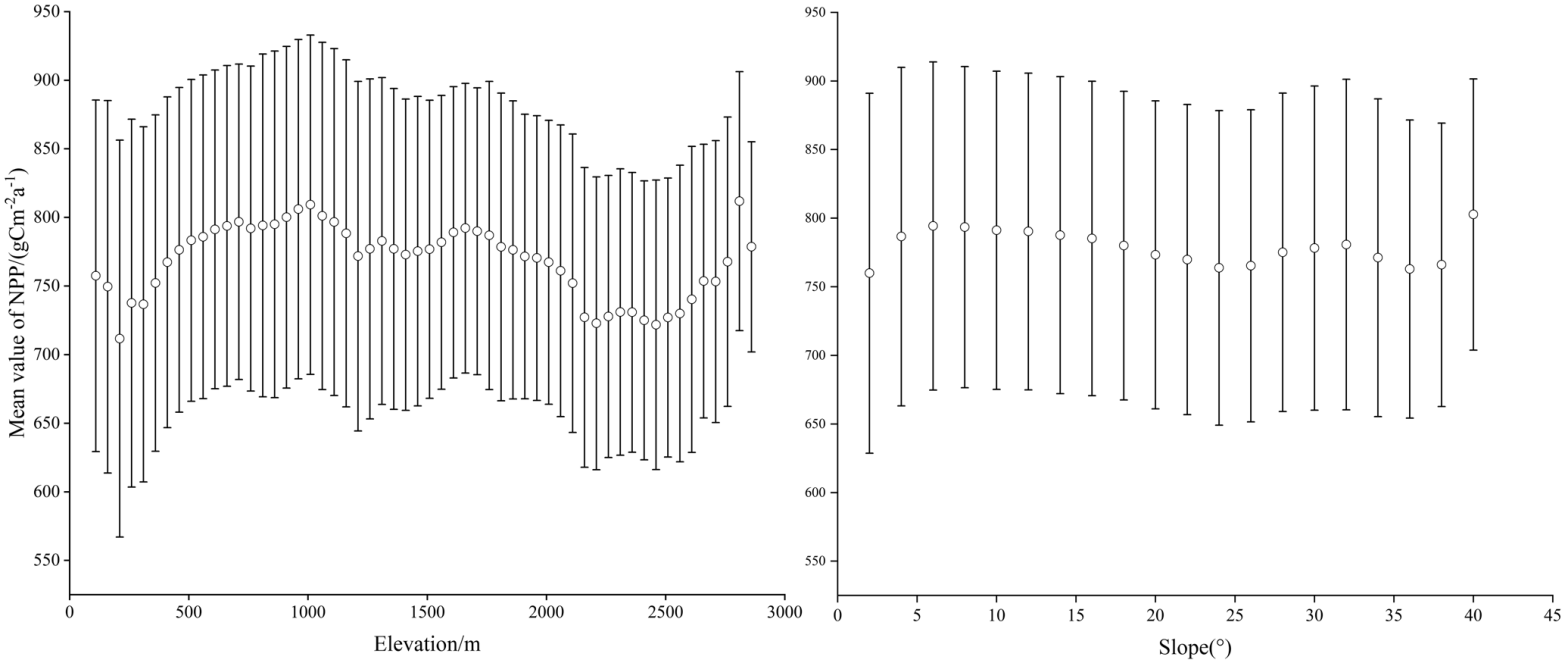
Change in NPP mean with elevation and slope.

In different topographical conditions, variations in temperature, moisture, and soil nutrients significantly influence vegetation growth, playing a crucial role in the accumulation and spatial distribution of NPP (Wang et al. 2023). To investigate the variation, we analyzed the data at intervals of 50 m and slope increments of 2 degrees, and the standard deviation represented the change amplitude (Figure 10). The annual average NPP fluctuation increased at an altitude of 807.84 g C m^−2^ a^−1^ at an altitude of less than 1000 m, decreased at 1000–2500 m, and increased above 2500 m. The lowest at 0°–3° was 759.89 g C m^−2^ a^−1^. The frequency of anthropogenic activities is higher in the lower slope area, and a large number of construction activities are carried out in this slope area, which leads to a low NPP value due to human disturbance. At 5°–25°, it shows a slow decline. At 25°–32°, NPP rises and then falls. While the slope is greater than 40°, the mean NPP for many years is the largest, which is 802.75 g C m^−2^ a^−1^.

#### 3.3.3 LULC Change

Through integrating land‐use transfer dynamics with NPP variations, we constructed a transition matrix to quantify their interdependencies.

As delineated in Table 5, land‐use conversions between grassland and forestland dominated NPP changes during 2000–2010. Conversions from other LULC to forestland contributed the largest NPP increment, with grassland‐to‐forestland transitions exhibiting the most pronounced enhancement. Concurrently, grassland and cultivated to other LULC predominated in NPP outflows. From 2010 to 2020, the transition from other LULC to grassland is the primary increase of NPP, while the shift from forest land to other LULC is also one of the primary reasons for the decrease of NPP. These spatiotemporal patterns highlight the critical role of ecological land‐use restructuring in modulating carbon sequestration capacity.

**TABLE 5:** Transfer matrix of NPP during 2000–2020 (10^−3^ *T g C*).

2000	2010					
	Cultivated land	Forest land	Grassland	Water	Construction land	Unused land
Cultivated land		174.93	44.58	−60.11	−28.03	0.04
Forest land	76.35		57.12	−50.66	−10.31	0.00
Grassland	68.72	350.74		−13.90	−4.90	−0.29
Water	0.61	0.26	0.44		0.01	0.00
Construction land	−0.73	−0.25	−0.12	−0.06		0.00
Unused land	0.24	1.20	−0.60	−0.19	−0.25	

2010	2020					
	Cultivated land	Forest land	Grassland	Water	Construction land	Unused land
Cultivated land		616.28	411.23	32.47	−86.23	0.51
Forest land	641.90		1693.46	85.55	−9.10	−0.22
Grassland	331.25	879.14		33.27	−17.57	0.27
Water	4.08	6.52	5.47		0.46	0.12
Construction land	5.93	5.60	3.48	2.67		0.00
Unused land	0.21	0.08	0.52	0.04	0.59	

#### 3.3.4 Quantitative Detection of the Driving Factors

**FIGURE 11:**
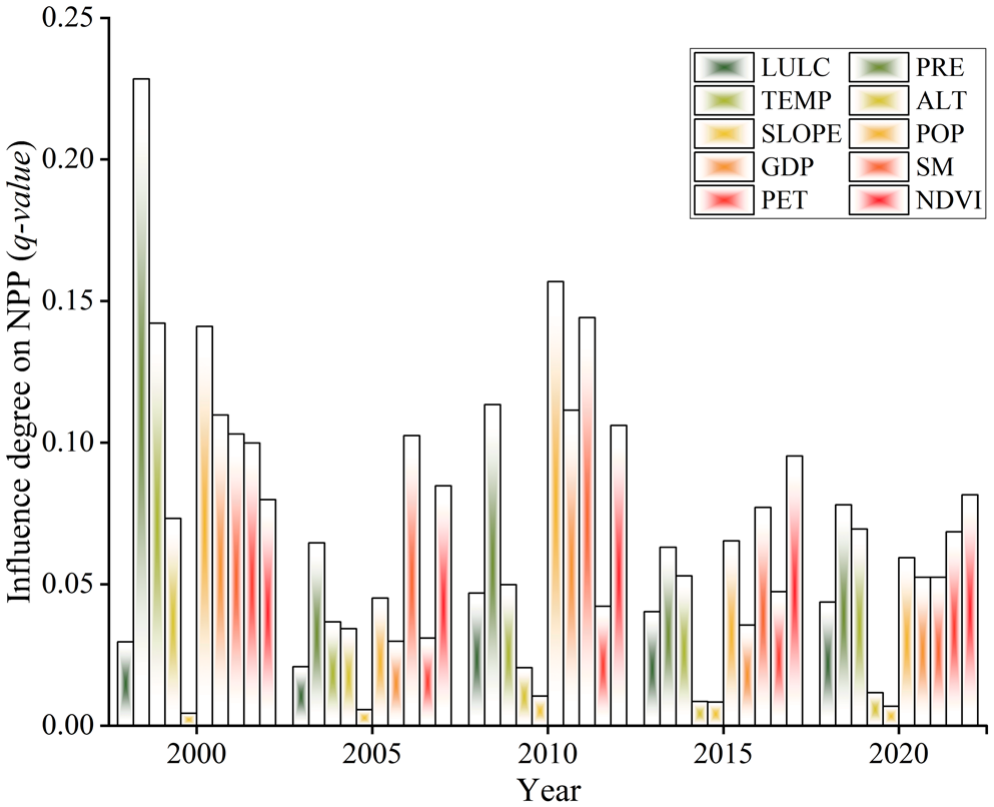
Results of different influencing factors on NPP in 2000, 2005, 2010, 2015, and 2020.

Different drivers have varying degrees of impact on the NPP in the same year, and the impact of the same factor on NPP differs across different years (Figure 11). In a horizontal comparison, in 2000, the top three driving factors were precipitation (0.228) > temperature (0.142) > population density (0.141). In 2005, they were soil moisture (0.102) > NDVI (0.085) > precipitation (0.065). In 2010, it was population density (0.157) > soil moisture (0.144) > precipitation (0.113). In 2015, NDVI (0.095) > soil moisture (0.077) > population density (0.065). In 2020, NDVI (0.081) > precipitation (0.078) > temperature (0.069).

**FIGURE 12:**
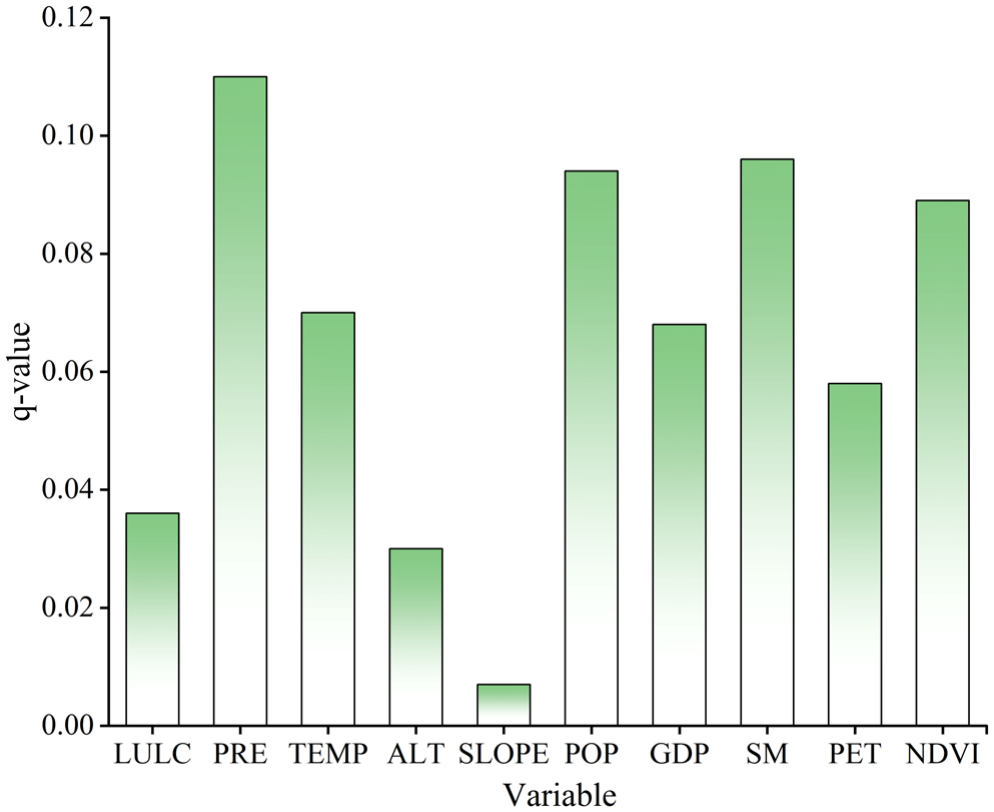
Mean values of the *q* values for the different influencing factors.

To further quantify the explanatory power of different driving factors on the spatial heterogeneity of NPP, the average *q* values of each driving factor in 2000, 2005, 2010, 2015, and 2020 were further calculated. The *q* values of the 10 selected drivers are precipitation, soil moisture, population density, NDVI, temperature, GDP, potential evapotranspiration, LULC, altitude, and slope in order of the 5‐year average size (Figure 12). The relatively large mean values belonged to precipitation, soil moisture, and population density, and their *q* values were 0.11, 0.096, and 0.094, respectively, collectively accounting for nearly 30% of NPP spatial variation in the past 20 years. As mentioned above, precipitation was the predominant driver leading to the heterogeneity of NPP in Guizhou province, but the impact of human activities began to become stronger over time.

**FIGURE 13:**
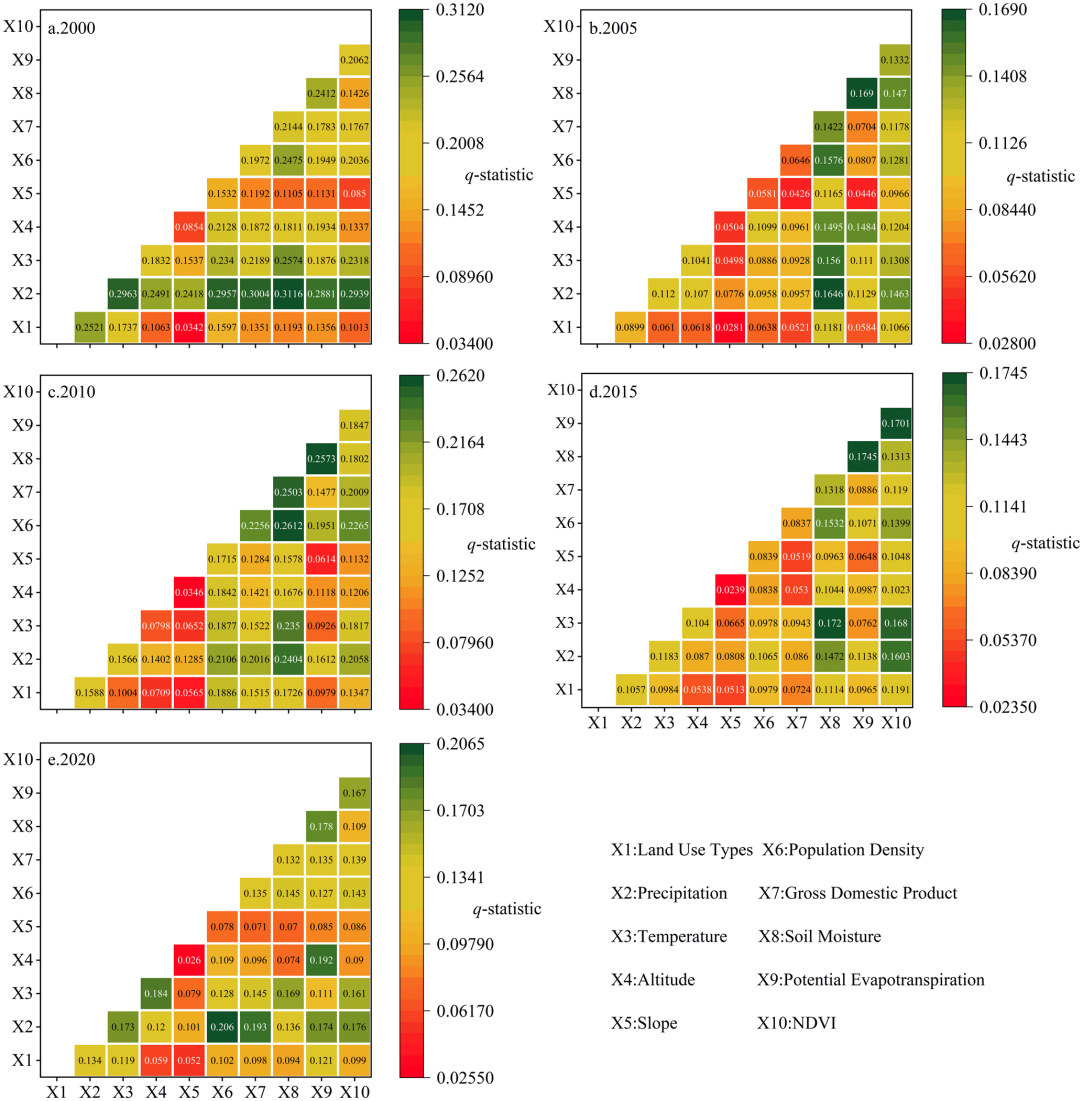
Interactive effect between influencing factors and NPP.

The interaction between the 10 driving factors is probed, including altitude, slope, GDP, population density, precipitation, soil moisture, temperature, potential evapotranspiration, LULC, and NDVI, and was significantly larger than that of the single factor (Figure 13). In 2000, the interaction between soil moisture and precipitation had the most obvious effect on vegetation NPP; in 2005 and 2015, the interaction between potential evapotranspiration and soil moisture was the strongest; in 2010; and in 2020, the interaction between population density and precipitation was the strongest. It can be seen that the interaction between factors significantly enhances the influence of single factors on vegetation, and no single factor can independently affect vegetation. This shows that the complex interaction between various factors is crucial in the process of vegetation growth and change.

## 4 Discussion

### 4.1 Impact of Precipitation and Temperature on NPP

The response of vegetation to the environment is a complex process that is affected by the coupling of multiple factors. The geographic detector revealed the dominant influence of natural factors, and water resources (precipitation, soil moisture) were the main climate drivers affecting NPP in Guizhou Province, which was similar to the results of the study (Qian et al. 2023; Wu et al. 2025). Previous studies have shown that water resources are an important constraint on vegetation growth in arid and semi‐arid regions (Shi et al. 2021). Despite Guizhou Province being a typical subtropical monsoon climate zone with affluent precipitation, Guizhou Province has continuous karst, high bedrock exposure, thin soil layers, low water storage capacity, and insufficient soil moisture, which are unable to meet the absorption and growth of plants in rock cracks and shallow soils (Zhu et al. 2025; Jiang et al. 2014; Jiang et al. 2022). Therefore, our research found that water resources are also a key limiting factor for vegetation growth in karst areas. The *q*‐value of the temperature is significantly lower than that of the hydrological factor, which confirms the low sensitivity of vegetation to thermal changes in subtropical regions (Chen et al. 2020). The mean temperature in Guizhou Province is 15.33°C, and the temperature is moderate. Elevated temperatures intensify potential evapotranspiration, thereby exacerbating soil moisture deficits during growing seasons (Running and Nemani 1991); however, a moderate increase in temperature promotes photosynthesis in vegetation.

### 4.2 The Impact of Human Activities on NPP

**FIGURE 14:**
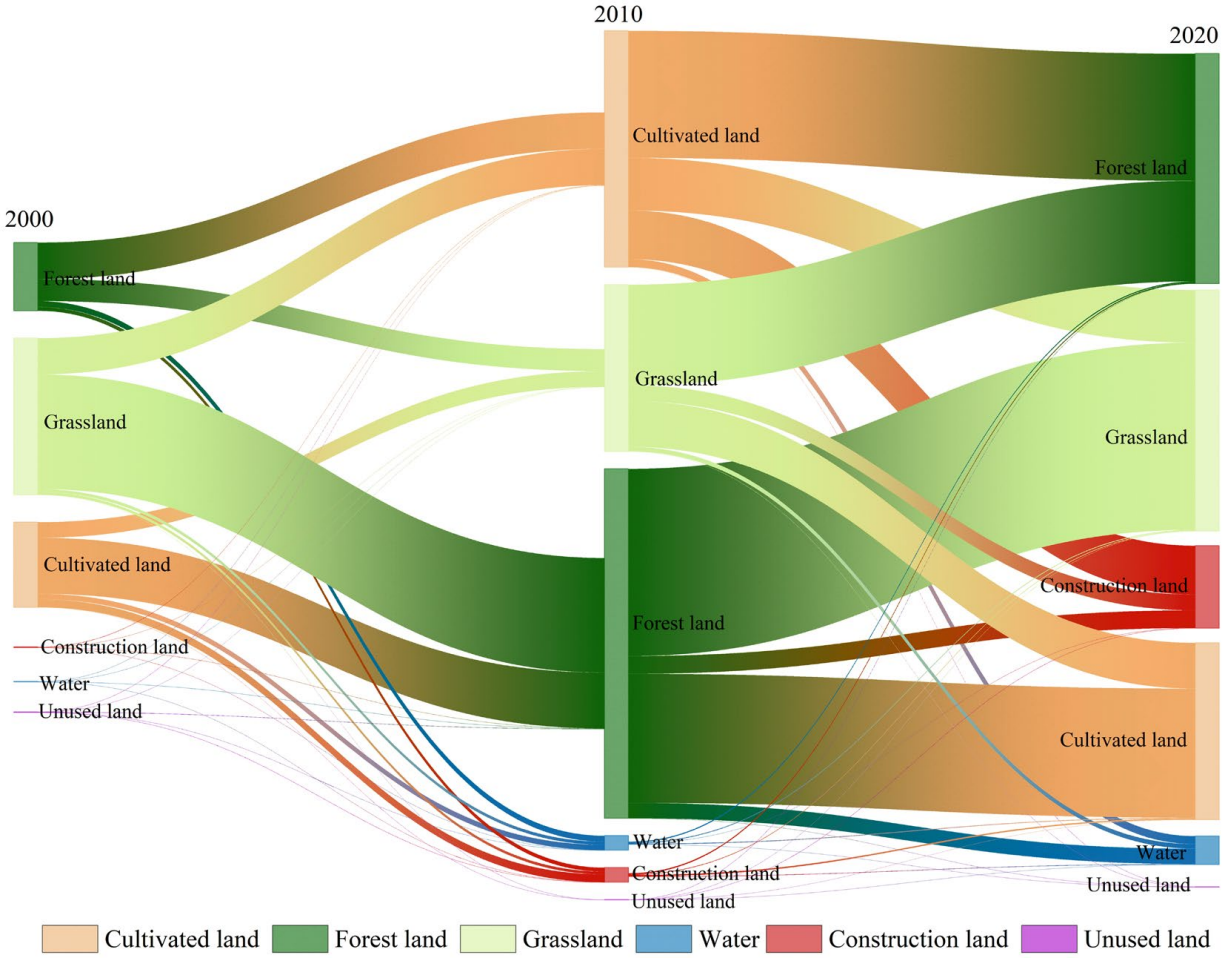
Sankey map of land cover transfer in Guizhou Province from 2000 to 2020.

LULC transformations dramatically alter the structure and functional integrity of native ecosystems, impacting both landscape patterns and carbon sequestration dynamics (Yang, Zhong, et al. 2021). Land‐use change represents a primary pathway through which human activities impact the natural environment, serving as a direct spatial manifestation of human activity intensity (Deng et al. 2024). By modifying land‐use patterns, human actions alter plant growth and distribution, consequently driving changes in vegetation NPP. The transfer from other LULC to construction land led to the decrease of NPP, and the construction land increased by 292.85% during the study period (Figure 14). Geographic detector results similarly show a trend of increasing anthropogenic impacts over time (Figure 11). With the rapid growth of the economy and population, urbanization is also advancing rapidly; the original vegetation cover is often destroyed, resulting in insufficient available space for plant growth, which adversely affects NPP (Chen et al. 2013; Ding et al. 2021; Ma et al. 2024). Of these, vegetation growth is particularly hindered in areas of high population density (Hao et al. 2021). The transitions from other LULC to forestland and grassland were the primary factors for the increase of NPP, and the transfer to construction land led to the decrease of NPP. The forestland was the dominant land type in Guizhou Province, accounting for more than 50%. Although the area decreased from 2000 to 2020, it increased by 1977.5 km^2^ from 2000 to 2010, which had a positive impact on NPP. The grassland area decreased by 2.7% in 2000–2020 but increased by 4.8% in 2010–2020, which is related to a series of ecological programs carried out in recent years. Since the implementation of the policy of returning farmland to forests at the beginning of the 21st century and the promotion of rocky desertification control projects after 2010, the trend of land desertification has been mitigated (Qiao et al. 2020). Since 2000, NDVI in China has increased at an average annual rate of 0.0021 (Wei, Sun, et al. 2022). Relevant studies have pointed out that the implementation of ecological projects has greatly promoted the vegetation ecology in most parts of China (Macias‐Fauria 2018; Zhang, Yue, et al. 2021) and has had a positive impact on NPP. This synergistic interplay between policy interventions and ecosystem recovery highlights the viability of targeted land management in balancing anthropogenic pressures and ecological sustainability. These results illustrate that ecological restoration projects contribute to vegetation greening in karst areas.

## 5 Conclusions

Based on multisource geospatial datasets, combined with Sen–Mann–Kendall trend analysis, coefficient of variation, Hurst index, and geographical detector model, this study systematically revealed the spatiotemporal variation and driving mechanisms of NPP in Guizhou Province from 2000 to 2020. The main conclusions were:

1. The annual average of NPP in Guizhou province increased significantly at the rate of 3.65 g C m^−2^ a^−1^, and the annual average was 785.47 g C m^−2^ a^−1^, with a typical spatial differentiation of “high in south and low in north,” 78.48% of the regional vegetation NPP showed an increasing trend.
2. The quantitative results of the geographic detectors show that water conditions are the primary driving force for the spatial heterogeneity of NPP, which confirms the “ecological water shortage” effect caused by karst thin soil layer and high leakage characteristics.
3. The dynamic enhancement of human influence, the expansion of construction land caused by urbanization, is the main cause of NPP loss, while the implementation of ecological engineering has a positive impact on the improvement of NPP.
4. The interaction between factors significantly enhances the influence of single factors on vegetation, breaking through the limitations of traditional linear models. Our study emphasizes that the geographical detector is an effective method to analyze the complex driving mechanism of vegetation change.

## Author Contributions

**Bo Xie:** conceptualization (lead), data curation (lead), formal analysis (lead), methodology (lead), writing – original draft (lead). **Yi Liu:** resources (supporting), software (supporting), writing – review and editing (supporting). **Han Fan:** resources (supporting), software (supporting), writing – review and editing (supporting). **Mingming Zhang:** funding acquisition (equal), project administration (equal), validation (equal).

## Conflicts of Interest

The authors declare no conflicts of interest.

## Data Availability

Code, NPP, Meteorological Data, Driving Factors data of the Guizhou Province in 2000 and 2020: Dryad Data Repository (http://doi.org/10.5061/dryad.gf1vhhn0w).
